# Diversity and dynamics of fungal endophytes in the roots of *Amomum villosum* lour. Under different areas and growth ages

**DOI:** 10.1186/s12866-025-04332-6

**Published:** 2025-08-26

**Authors:** Xiao-Gang Li, Xiao-Xu You, Xing-Kai Zhang, Di Chen, Sen He, Guan-Hua Cao

**Affiliations:** 1 School of Chinese Materia Medica and Chinese Pharmaceutical Research International Science and Technology Cooperation Base of Yunnan University of Chinese Medicine, Kunming, 650500 Yunnan China; 2 Wenshan Inspection of Food and Drug Control, Wenshan, 663099 Yunnan China; 3 State Key Laboratory for Quality Ensurance and Sustainable Use of Dao-di Herbs, Beijing, 100700 China

**Keywords:** Medicinal plant, Endophytic fungi (EF), Colonization rate, Fungal community composition, Dark septate endophyte (DSE), Arbuscular mycorrhizal fungi (AMF)

## Abstract

This study aims to investigate the colonization and diversity of endophytic fungi in *Amomum villosum* roots across different planting locations and at various growth ages, and to analyze the fungal composition. In this study, we performed Illumina-based ITS sequencing to investigate the effects of growth ages and sample plots on the rhizosphere fungi of *A. villosum*. Colonization analysis revealed widespread colonization by arbuscular mycorrhizal fungi (AMF) and dark septate endophytes (DSEs), but colonization rates did not increase significantly with plant age. *Glomus* was the dominant AMF genus, while *Exophiala*, *Cladosporium* and *Cladophialophora* dominated the DSE community. Beneficial fungi included *Phoma*, *Acremonium*, *Myrothecium* and *Trichoderma*. Alpha and beta diversity analyses indicated that fungal diversity, abundance, and community composition were significantly influenced by planting location but not by plant age. Collectively, planting location drives divergent root fungal communities in *A. villosum*, while plant age selectively affects specific taxa. AMF and DSEs were considered to be dominant beneficial microorganisms and were largely responsible for the growth and development of *A. villosum*. This study provides valuable insights for identifying beneficial endophytic fungi in *A. villosum* and optimizes the application of AMF and DSE agents in its ecological cultivation.

**Supplementary Information:**

The online version contains supplementary material available at 10.1186/s12866-025-04332-6.

## Introduction

Microorganisms critically mediate plant-environment interactions during long-term growth. Healthy plants harbor diverse microbial communities within roots, stems, and leaves, collectively termed the plant microbiota [1, 2]. As primary entry points for soil microorganisms, roots accumulate abundant endophytic fungi (EF) spanning Ascomycota, Basidiomycota, and Glomeromycota [3]. Among these, arbuscular mycorrhizal fungi (AMF) and dark septate endophytes (DSEs) establish intracellular symbioses that enhance nutrient acquisition and stress tolerance [4, 5]. These symbioses rely on carbon transfer from host plants, reciprocated by fungal provision of nutrients via signaling molecules such as transporter proteins, bioactive compounds, and mycorrhizal factors (Myc factors) [6, 7]. Myc factors-derived from AMF are recognized by host roots and essential for establishing functional symbioses [8]. Although AMF enhance plant resilience to drought, salinity, heavy metals, and phosphorus deficiency [9, 10, 11, 12], their mechanisms require further elucidation. Understanding complex microbe-microbe and microbe-plant interactions is critical for addressing agricultural challenges and developing solutions for crop cultivation and ecosystem management. Root endophytic fungal communities exhibit complex compositional dynamics influenced by plant health status, developmental stage, growth environment of the plant. Huang et al. [13] reported similar fungal communities in healthy and diseased *Eustoma grandiflorum* root but noted enrichment of disease-suppressive fungi in healthy plants versus pathogens in diseased ones. Šmilauer et al. [14] and Kil et al. [15] observed reduced AMF diversity in juvenile grasses due to limited carbon allocation to symbionts. Soil nutrient profiles and geographic location also significantly modulate EF communities [12, 16].

While pure culture isolation remains an effective method for studying cultivable EF, many taxa-particularly AMF-resist cultivation due to biological uncultur ability [17, 18]. In contrast, Illumina sequencing of the internal transcribed spacer (ITS) region enables community-wide profiling of EF diversity at the molecular level. This approach detects unculturable species and reveals functional relationships within fungal communities, thereby addressing fundamental questions about ecosystem functions [19, 20].

*Amomum villosum* Lour. is a perennial medicinal herb of the genus *Amomum* in the Zingiberaceae family, with a long history of usage [21]. Its fruits possess various medicinal properties [22]. Distributed in humid mountainous regions of Yunnan, Guangdong, and Hainan (China), *A. villosum* thrives in humus-rich, shaded forest soils [21, 23]. Prior studies suggest complex rhizosphere microbiota influence its growth, yield, and fruit quality [24], but root EF symbionts remain uncharacterized. Thus, we urgently require a systematic investigation of *A. villosum* root EF. Characterization of colonization patterns and molecular profiling of *A. villosum* root EF are required to elucidate their diversity. This study employed high-throughput sequencing to investigate fungal community structure and diversity within *A. villosum* roots and to assess variations associated with distinct geographical locations and plant developmental stages.

## Materials and methods

### Sample collection

**Fig. 1:**
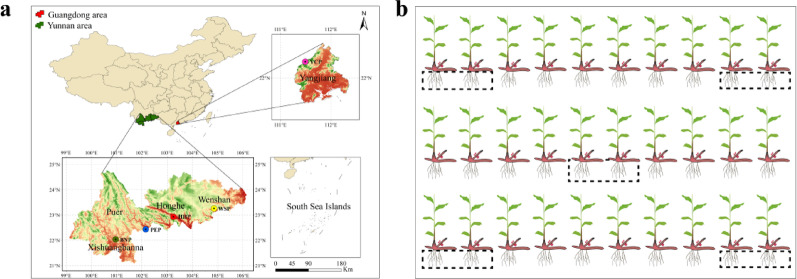
Distribution map of sampling plots (**a**) and sampling method diagram (**b**). *A. villosum* samples were harvested from five plots: Xishuangbanna (BNP), Pu’er (PEP), Honghe (HHP) and Wenshan (WSP) in Yunnan, and Yangchun (YCP) in Guangdong, which are the main production regions in China (**a**). A five-point sampling method was used to collect samples, and ten plants of each age group in each plot were sampled (**b**)

As shown in Fig. 1a, A. *villosum* samples were collected from its primary production regions in southern China: Yunnan and Guangdong Provinces. In September 2021, roots were harvested from five plots: Xishuangbanna (BNP), Pu’er (PEP), Honghe (HHP), and Wenshan (WSP) in Yunnan, and Yangchun (YCP) in Guangdong. The geographic coordinates of sampling points and agro-meteorological data are provided as a supplementary Table S1.

*A. villosum* samples consisted of two age groups of one and three years old, which were newly sprouted plants in 2020 and planted in 2018. The first and second year after transplanting is growth stage, during which the *A. villosum* cannot produce fruit normally or has a low fruit rate, and flower and bear fruit normally in the third year and later. Therefore, annual and triennial samples of *A. villosum* roots were selected as research materials. Ten sample groups (BNP-1, BNP-3, PEP-1, PEP-3, HHP-1, HHP-3, WSP-1, WSP-3, YCP-1, YCP-3), were analyzed. Per plot, both age groups were collected via a five-point sampling method (Fig. 1b), yielding ten plants per age group. During sampling, the two diagonal lines of the sample plot were determined first, and the intersection point is selected as the central sampling point, four equidistant points were selected along the diagonals. Fresh fibrous roots were transported to the laboratory, repeatedly washed with sterile water to remove soil particles, then pooled by group. Each pooled sample (three biological replicates) was divided into two aliquots in 50 mL sterile EP tubes: one for sequencing (Ronin Bio, China), and one stored at -80 °C for further analysis [25].

### Assessment of fungal colonization

All fibrous roots were thoroughly cleaned and stained according to Liu et al. [9]. Fresh roots were sectioned into 1 ~ 1.5 cm segments, then soaked in 10% (w/v) KOH at 90 ℃ for 60 min. After rinsing with tap water, root segments were stained in a boiling solution of 0.05% Parker Quink ink in lactophenol for 3 min. Subsequently, stained root segments were cleared with 5% (v/v) acetic acid and mounted in lactic acid-glycerol on microscope slides following squashing. Root endophyte colonization was quantified at 40× magnification using a compound microscope (Nikon, Eclipse E100) via the magnified intersection method. For each 1.5 cm root segment, approximately 100 fields of view were examined. Colonization rates of AMF and DSEs were calculated based on the presence of characteristic structures: for AMF: vesicles, arbuscules, and hyphal circles; for DSEs: dark myceliums, septate hypha, and microsclerotia [9].

### DNA extraction, PCR amplification, and sequencing

Genomic DNA from root fungi was extracted and purified using the Zymo Research BIOMICS DNA Microprep Kit (Zymo Research, USA) according to the manufacturer’s protocol. The fungal ITS region was amplified via polymerase chain reaction (PCR) with primers ITS3 (5’-GATGAAGAACGYAGYRAA-3’) and ITS4 (5’-TCCTCCGCTTATTGATATGC-3’) [26]. Each sample was amplified in triplicate in a 25 µL system containing 1 U KOD-Plus-Neo polymerase, 1× PCR Buffer for KOD-Plus-Neo, 0.2 mM dNTPs, 1.5 mM MgSO_4_, and 0.3 µM forward and reverse primers, 20 ng/µL template DNA, and GSS Depletion Mix (Rhonin Bio, China) to remove interference from the elimination of chloroplast and mitochondrial sequences in the samples. Amplification was performed with an Applied Biosystems^®^ PCR System 9700 (USA) according to the following conditions: 94 °C/1 min for one cycle; 94 °C/20 s, 54 °C/30 s and 72 °C/30 s for 25–30 cycles; and 72 °C/5 min for one cycle. Afterward, the PCR products were electrophoresed on 2% agarose gels, purified, and quantified.

### Library construction and sequencing

Library construction was performed using the NEBNext Ultra II DNA Library Prep Kit for Illumina (BioLabs Inc., New England, USA). Subsequently, purified amplicons were pooled in equimolar ratios and subjected to paired end sequencing (2 × 250 bp) using an Illumina MiSeq platform (Illumina, San Diego, USA) following standard protocols at Ronin Bio (China).

### Data processing and community composition analysis

Merged double-ended sequences were obtained as raw tags. These tags underwent quality filtering via Quantitative Insights Into Microbial Ecology (QIIME, v1.8.0, http://qiime.org/) to generate high-quality clean tags. Subsequently, chimeras were removed using the UCHIME algorithm [27, 28]. Based on the Usearch software (http://drive.com/uparse/), operational taxonomic units (OTUs) were clustered at a 97% sequence identity threshold using the UPARSE algorithm within Usearch software [29]. The most abundant sequence within each OTU was selected as its representative sequence and annotated using the UNITE database (version 7.0) [30]. OTU abundance data was normalized to the sample with the fewest sequences. Alpha and beta diversity analyses were performed using these normalized data. Community composition was visualized with the ggplot2 package in R. Alpha diversity indices (Chao1, ACE, Shannon, Simpson, Good’s coverage) were calculated using the vegan package in R, while Faith’s Phylogenetic Diversity (PD) was calculated with the picante package [31]. Differences in parameter variance among samples from different sample plots were estimated by ANOVA. Multiple comparisons were performed using the agricolae package. Differences between annual and triennial samples were conducted using the Wilcoxon rank sum test (Wilcox. test, stats package). Principal coordinates analysis (PCoA, GUniFrac package in R language) was performed using a weighted UniFrac distance index to detect similarity between samples [32]. To test the effect of spatiotemporal factors on community structure, PERMANOVA (adonis function in the vegan package) was applied to explain the significance of temporal and spatial effects on sample differences at a significance level of *p* < 0.05. Key discriminatory genera across groups were identified via combined Random Forest analysis and differential abundance testing.

## Results

### Colonization rates of AMF and DSEs in *A. villosum* roots

**Fig. 2:**
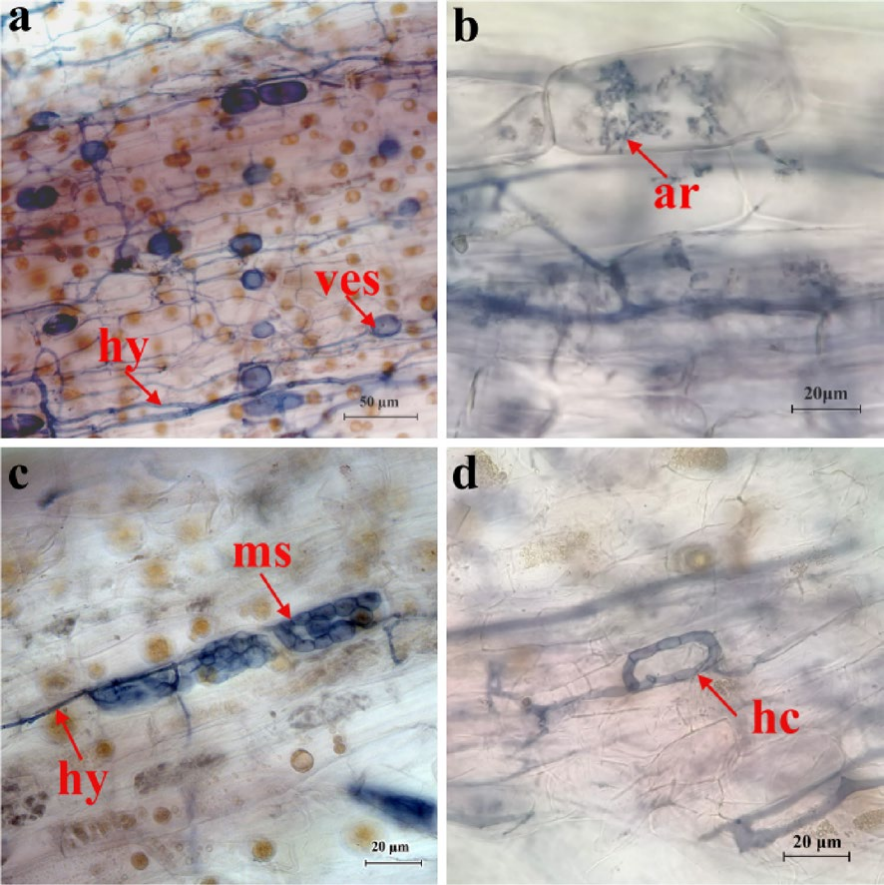
Fungal root symbionts in *A. villosum* roots. **a**, Vesicles (ves) and hypha (hy) of AMF; **b**, Arbuscules (ar) of AMF; **c**, Microsclerotias (ms) and hypha (hy) of DSEs; **d**, Hyphal circle (hc) of AMF. The typical structures of AMF and DSEs were widely observed in the intracellular or intracellular space of *A. villosum* roots. Scale bars in Fig. 2**a** and the other three figures (**b**, **c** and **d**) are 50 and 20 μm, respectively

Typical structures of AMF and DSEs were observed in the intracellular and intracellular space of *A. villosum* roots, including AMF hypha (hy), vesicles (ves) and arbuscules (ar) and DSE microsclerotia (ms) with septate hypha (hy) (Fig. 2). The presence of hypha, arbuscules, microsclerotia and septate hypha signaled the establishment of symbiotic associations facilitating the exchange of carbon and nutrients. In annual samples, AMF colonization rates were highest at HHP, WSP and PEP plots (34.29 ± 5.02, 32.39 ± 8.72, and 28.72 ± 5.07%, respectively), followed by YCP (10.07 ± 4.74%) and BNP (3.87 ± 3.38%) (Table 1). Colonization rates in BNP, WSP and YCP plots increased after three years of growth, with the triennial YCP sample exhibiting a 251.44% increase. However, arbuscular colonization rates were lower than those of other structures (e.g., hypha and vesicles), especially in BNP-1, YCP-1, BNP-3, and PEP-3 (< 1%). DSEs colonization rates were consistently lower than those of AMF across samples. The annual PEP samples exhibited the largest DSEs colonization rate (9.14 ± 4.73%), while no DSEs structures were observed in the annual BNP sample. Relative to annual samples, triennial BNP, HHP, and YCP samples exhibited higher colonization rates, whereas WSP and PEP showed either declines or no significant changes.

**Table 1:** Summary of the colonization rates of AMF and DSEs

Groups	AMF colonization rates (%)				DSE colonization rates (%)		
	Total	Hypha	Vesicles	Arbuscules	Total	Septate hypha	Microsclerotia
BNP-1	3.87 ± 3.38^c^	3.70 ± 3.21^b^	0.31 ± 0.24^d^	0.26 ± 0.28^b^	0^b^	0^b^	0^c^
PEP-1	32.39 ± 8.72^a^	28.71 ± 7.97^a^	14.01 ± 5.89^b^	1.87 ± 1.45^a^	9.14 ± 4.73^a^	8.88 ± 4.65^a^	0.45 ± 0.84^bc^
HHP-1	34.29 ± 5.02^a^	31.64 ± 5.39^a^	21.17 ± 6.81^a^	1.50 ± 0.79^a^	1.92 ± 1.59^b^	1.21 ± 0.97^b^	0.97 ± 1.17^a^
WSP-1	28.72 ± 5.07^a^	27.35 ± 6.25^a^	7.85 ± 3.97^c^	1.25 ± 0.80^a^	2.94 ± 3.31^b^	2.51 ± 3.21^b^	0.67 ± 1.02^bc^
YCP-1	10.07 ± 4.74^b^	1.54 ± 1.87^b^	9.41 ± 4.27^bc^	0.16 ± 0.30^b^	1.59 ± 1.92^b^	0.87 ± 1.36^b^	0.84 ± 1.43a^b^
BNP-3	13.03 ± 6.93^c^	12.15 ± 6.85^c^	5.26 ± 4.13^c^	0.60 ± 0.47^a^	6.57 ± 4.57^a^	3.97 ± 3.09^bc^	5.21 ± 4.28^a^
PEP-3	22.79 ± 6.16^b^	21.32 ± 6.41^b^	8.02 ± 5.20^c^	0.72 ± 0.75^a^	9.38 ± 5.83^a^	9.51 ± 6.06^a^	0.19 ± 0.26^b^
HHP-3	24.45 ± 2.88^b^	20.44 ± 3.25^b^	8.73 ± 3.14b^c^	1.45 ± 0.72^a^	6.83 ± 5.9^a^	7.60 ± 2.96^ab^	4.80 ± 2.59^a^
WSP-3	39.48 ± 4.93^a^	39.52 ± 10.08^a^	16.02 ± 7.20^b^	1.41 ± 1.06^a^	0.89 ± 1.02^b^	0.37 ± 0.58^c^	0.65 ± 0.97^b^
YCP-3	35.39 ± 6.02^a^	1.45 ± 3.34^c^	37.17 ± 9.54^a^	1.38 ± 1.24^a^	7.05 ± 6.74^a^	0.80 ± 1.28^c^	6.31 ± 7.79^a^

Different lowercase characters indicate statistically significant differences among groups, *p* ≤ 0.05

### Sequencing results

A total of 978,191 high-quality clean tags were obtained (29,990 ~ 38,943 reads per sample). After UCHIME-based chimera removal, effective tags were obtained for subsequent analysis with an average length of 341 bp, in the range of 335 ~ 352 bp (Table S2). Clustering at 97% identity yielded 1,092 fungal OTUs, annotated to 9 phyla, 26 classes, 69 orders, 149 families, 254 genera and 270 species.

### Composition of endophytic fungal communities

**Fig. 3:**
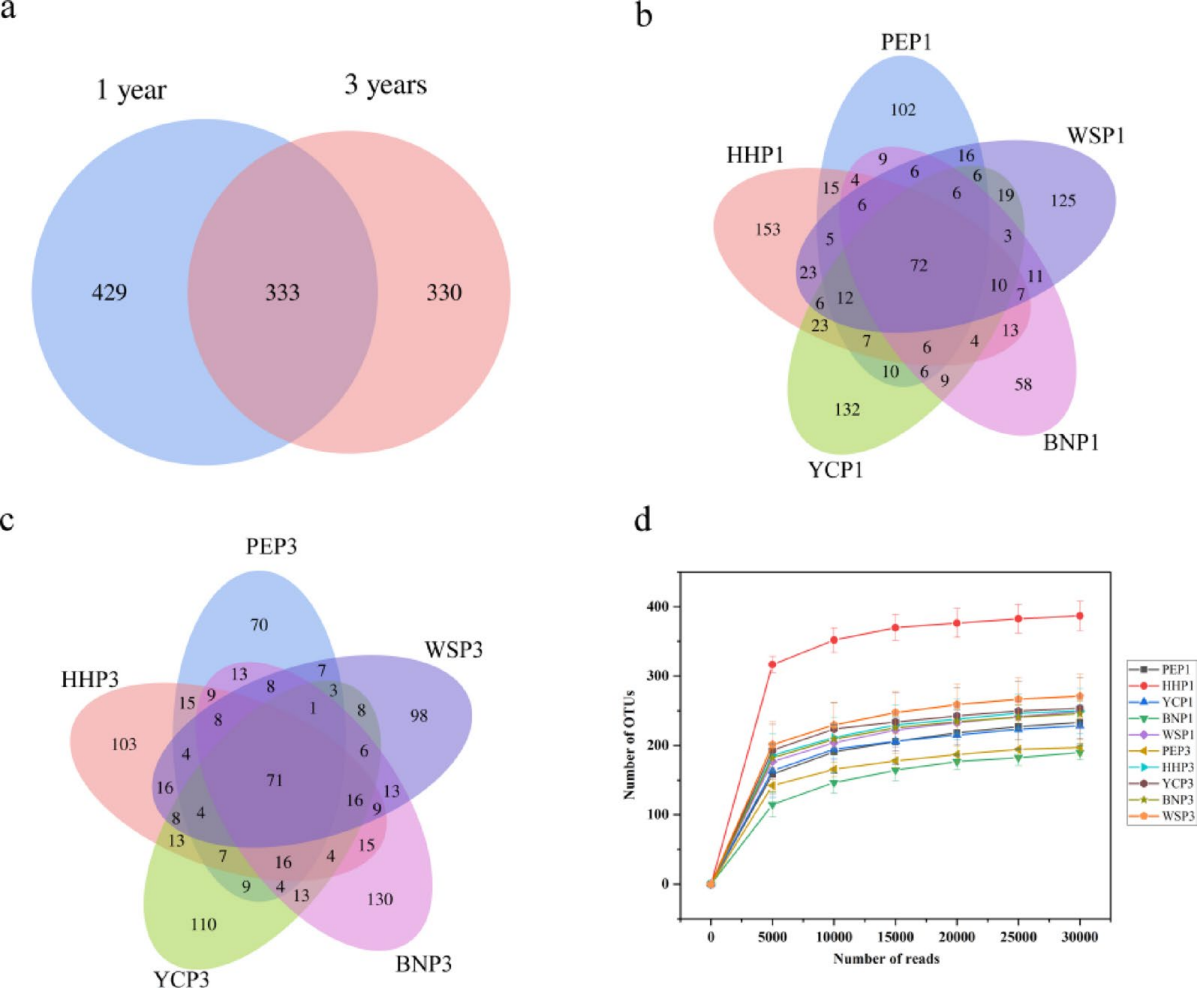
Venn diagrams and rarefaction curves for samples based on the number of shared OTUs. **a**, Number of OTUs shared between annual and triennial samples; **b**, Number of OTUs shared among different provenances of annual samples; **c**, Number of OTUs shared among different provenances of triennial samples; **d**, Rarefaction curves of all samples. A total of 333 OTUs were shared between annual and triennial samples, accounting for a proportion of 30.49% (**a**). There were 72 and 71 common OTUs among the annual or triennial samples, respectively (**b**, **c**). The rarefaction curves showed that the annual HHP samples had higher OTUs than other samples (**d**)

Venn analysis revealed 333 shared OTUs between annual and triennial samples (30.49% of total; Fig. 3a), with 429 (39.29%) and 330 (30.22%) unique to annual and triennial samples, respectively, indicating decreased diversity of EF in *A. villosum* roots with plant age. For the annual samples, excluding 72 common OTUs, the OTUs specific to each group were ranked as HHP (153) > YCP (132) > WSP (125) > PEP (102) > BNP (58) (Fig. 3b), and the sort order was BNP (130) > YCP (110) > HHP (103) > WSP (98) > PEP (70) for triennial samples without 71 common OTUs (Fig. 3c). Rarefaction curves showed the highest OTU richness in annual HHP and lowest in annual BNP samples (Fig. 3d).

**Fig. 4:**
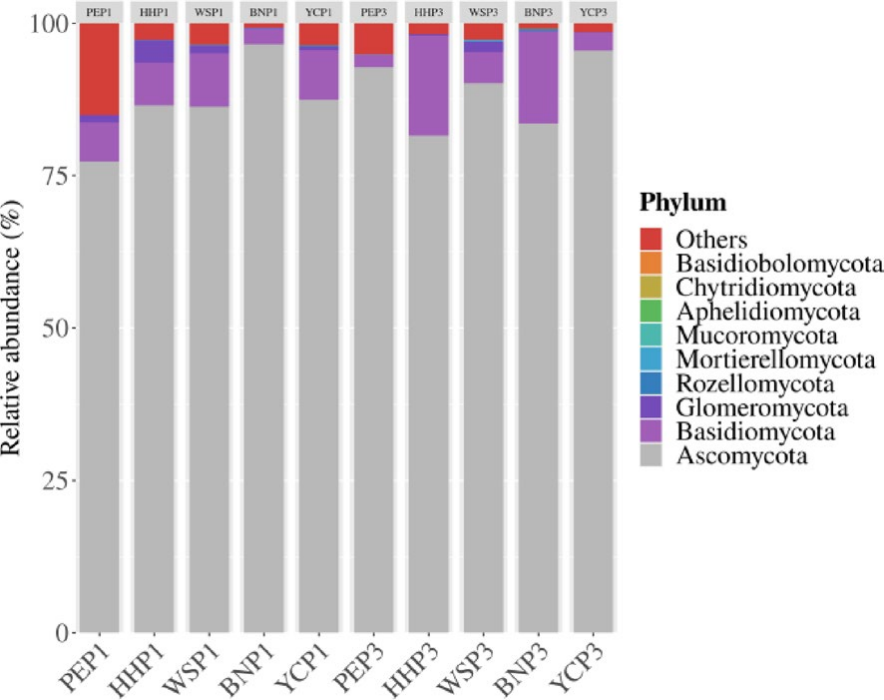
Phylum-level distribution of fungi in the root system of *A. villosum* samples. Ascomycota was the predominant phylum for annual and triennial samples with ratios of 93.58 and 92.39%, respectively, followed by Basidiomycota (2.96% and 5.75%)

Ascomycota dominated both annual (93.58%) and triennial (92.39%) samples, followed by Basidiomycota (2.96% and 5.75%; Fig. 4). Glomeromycota (AMF-associated) constituted 0.77% (annual) and 0.22% (triennial). Sordariomycetes was the dominant class (81.88% annual and 71.77% triennial), with Dothideomycetes 7.61% was higher in triennial samples. Dominant orders included Pleosporales (13.00% annual and 19.53% triennial) and the EF order Hypocreales (66.63% annual and 50.53% triennial) (Figure S1). Co-dominant families were Cordycipitaceae (51.66% annual and 15.92% triennial) and Nectriaceae (8.28% annual and 7.15% triennial). In addition, Cladosporiaceae abundance increased significantly from 0.21% (annual) to 3.15% (triennial), which was usually supposed to be a growth-promoting microorganism.

**Fig. 5:**
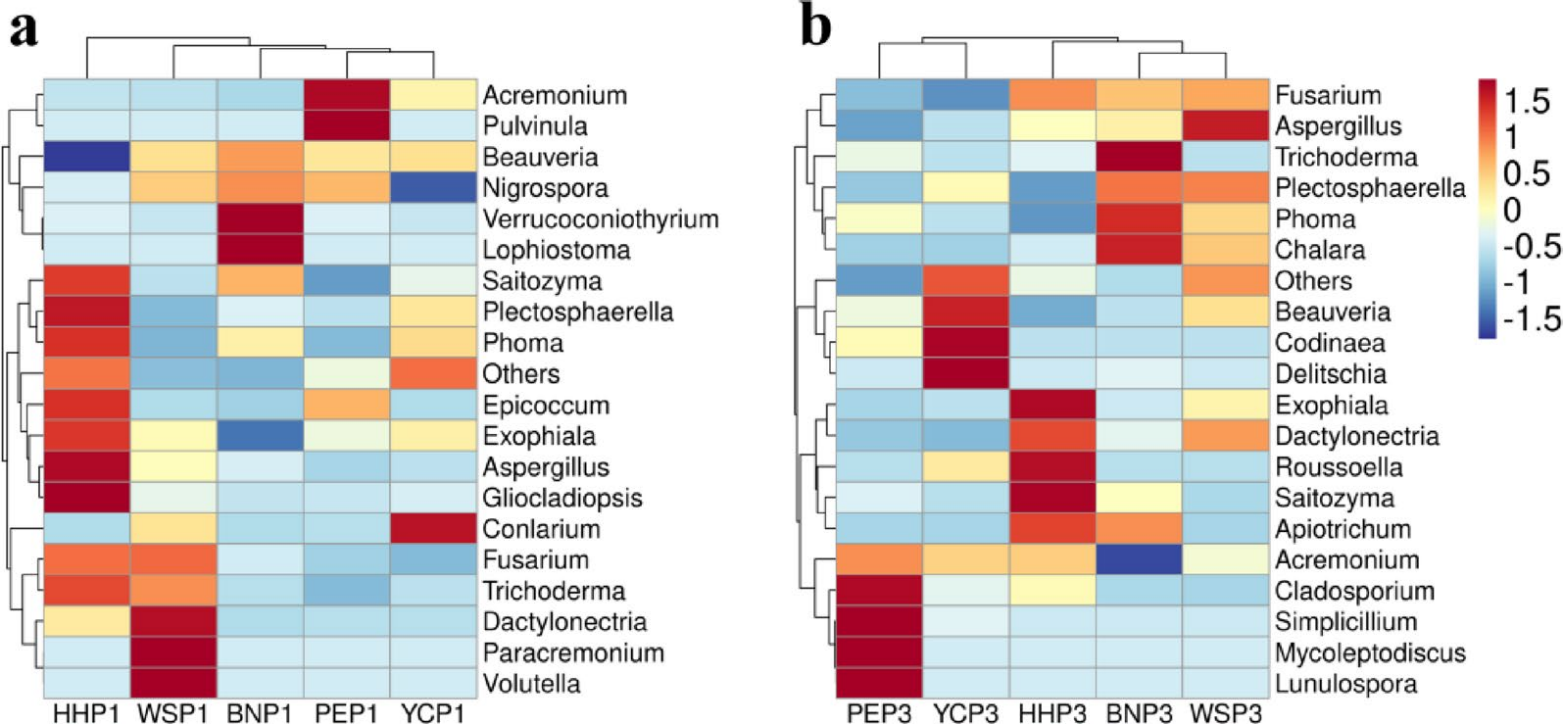
Heatmap analysis of root endophytic fungal communities in *A. villosum* across planting locations and plant ages (top 20 genera). **a** and **b** represent the annual and triennial samples, respectively. The genera *Beauveria* (51.47%/35.05%, one-year/three-year) and *Fusarium* (6.43%/8.52%) were identified as the main codominant genera in annual and triennial samples. The potential antimicrobial fungi *Phoma* (1.65%/4.32%), the saprophytic fungi *Acremonium* (3.87%/2.24%) and *Myrothecium* (0.09%/0.02%), and the biocontrol fungi *Trichoderma* (0.88%/1.04%) were major genera for all samples

At the genus level, the top 20 most abundant genera were selected to generate mapping images separately (Fig. 5). Co-dominant taxa included *Beauveria* (51.47% annual, 35.05% triennial) and *Fusarium* (6.43% annual, 8.52% triennial). The potential antimicrobial fungi *Phoma* (1.65% annual, 4.32% triennial), the saprophytic fungi *Acremonium* (3.87% annual, 2.24% triennial) and *Myrothecium* (0.09% annual, 0.02% triennial), and the biocontrol fungi *Trichoderma* (0.88% annual, 1.04% triennial) were major genera for all samples in this study. The proportions of *Exophiala*, *Cladosporium* and *Cladophialophora* increased to 0.22 ~ 1.18%, 0.18 ~ 3.05%, and 0.15 ~ 0.25%, respectively, of which these genera could be classified as DSE in taxonomic status. It is speculated that these fungi play an important role in the succession of *A. villosum*. In addition, *Glomus* (0.12% annual, 0.04% triennial) was the dominant genus of AMF but ranked below 20 in abundance. The EMF fungus *Tomentella* and DSE *Phialocephala* were found only in annual samples, and the saprophytic fungus *Oidiodendron* was found alone in triennial samples.

### The response of the alpha diversity of root EF to Temporal space

**Fig. 6:**
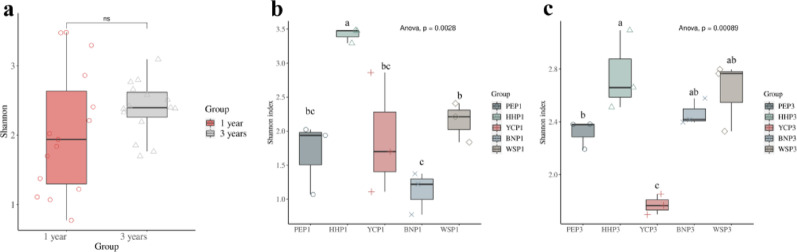
Alpha-diversity of EF communities in *A. villosum* roots across planting locations and plant ages. **a**, Comparison of annual and triennial samples. **b**, Annual samples from 5 sampling plots. **c**, Triennial samples from 5 sampling plots. The fungal community richness and diversity of the annual samples were not significantly different from those of the triennial samples (**a**). Among the samples from the 5 sample plots, the annual and triennial samples of the HHP plots had the highest richness and diversity and showed a significant decrease in diversity and richness after three years of growth. In contrast, the diversity and richness of the PEP, BNP and WSP samples increased significantly with increasing plant age (**b**, **c**). Different lowercase characters above the bars indicate significant differences among samples (*p* ≤ 0.01). Bars indicate mean ± SEM (*n* = 3)

Community richness and diversity were assessed using five indices: Chao1, Shannon, Simpson, ACE, and Good’s coverage. Sequencing depth (Good’s coverage) exceeded 99% (99.8–100%) for all samples, indicating sufficient sampling depth (Table S3). Chao1 and ACE indices reflect fungal community richness, while Shannon and Simpson reflect diversity. The fungal community richness and diversity of the annual root samples were not significantly different from those of the triennial samples (Fig. 6a). Among the five sample plots, HHP exhibited the highest initial richness and diversity in both annual and triennial samples but showed significant reductions after three years. Conversely, PEP, BNP, and WSP samples displayed significant increases in diversity and richness with plant age (Fig. 6b, c).

### Response of beta diversity of root EF to Temporal space

**Fig. 7:**
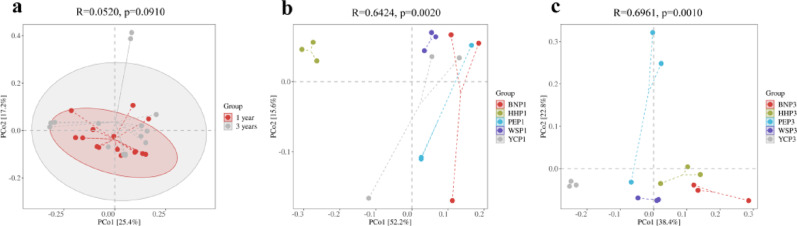
Principal coordinate analysis for root endophytic fungal communities in *A. villosum* across planting locations and plant ages. **a**, Comparison between annual and triennial samples. **b**, Annual samples from 5 sampling plots. **c**, Triennial samples from 5 sampling plots. *A. villosum* root endophytic fungi community composition was not significantly different between samples of two growth ages but rather presented among samples of different sampling plots (**a**). Independent analyses showed significant differences in fungal community composition among the five geographically located annual samples, and triennial samples also differed significantly by geographic location (**b**, **c**)

Weighted UniFrac PCoA revealed the *A. villosum* root EF community composition significant differences among geographic plots, but not between plant ages (Fig. 7a). PERMANOVA analyses showed significant differences in fungal community composition among the five geographic plots for both annual samples (*R* = 0.6424, *P* = 0.0020), and triennial samples (*R* = 0.6961, *P* = 0.0010) (Fig. 7b, c).

### Differences in fungal communities

**Fig. 8:**
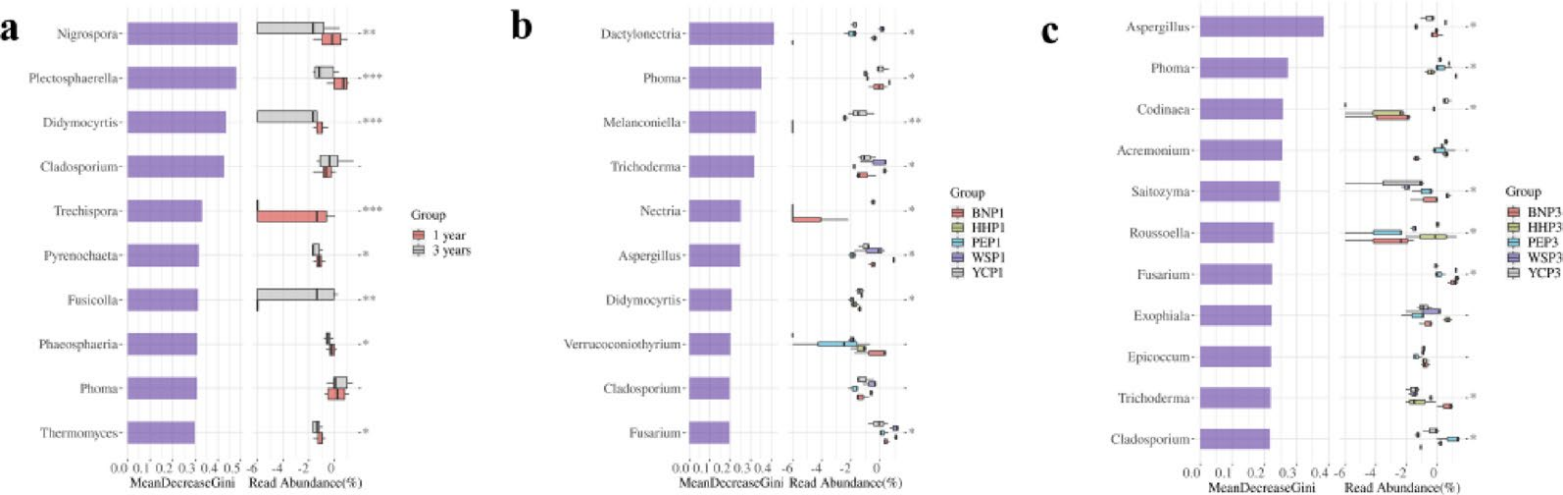
Random forest biomarker analysis of *A. villosum* root endophytic fungal communities across planting locations and plant ages. **a**, Comparison between annual and triennial samples. **b**, Annual samples from 5 sampling plots. **c**, Triennial samples from 5 sampling plots. *Nigrospora* and *Cladosporium* were the main biomarkers in the annual and triennial samples, respectively. The abundance of *Nigrospora* significantly increased with plant ages while the abundance of *Cladosporium* differed significantly among groups of five triennial samples (**a**). Species of *Trichoderma* were significantly enriched in the annual samples, and *Aspergillus* species were dominant in the triennial samples (**b**, **c**)

To determine the taxonomic fungal taxa with significant differences in abundance between annual and triennial samples, random forest analysis was used to detect biomarkers, in which a higher Gini index indicated that the fungus was more taxonomically important between groups (Fig. 8). *Nigrospora* and *Cladosporium* were identified as key biomarkers for annual and triennial samples, respectively. The abundance of *Nigrospora* significantly increased with plant age while the abundance of *Cladosporium* differed significantly among the triennial groups. *Trichoderma* species were significantly enriched in annual samples, whereas *Aspergillus* species dominated triennial samples. Both genera showed significantly higher abundance than other taxa within their respective plot samples.

## Discussion

In this study, we evaluated the EF colonization of *A. villosum* roots under different planting locations and growth ages for the first time. High-throughput sequencing technology was employed to analyze the abundance, composition and distribution of root EF communities.

AMF and DSEs are the two main groups of EF and play critical roles in the growth and development of host plants. Numerous studies demonstrate that AMF promote plant growth and development under different habitats and nutritional conditions, particularly by enhancing phosphorus uptake in the root system [33]. It is established that DSEs possess similar functions, although further evidence is required to elucidate their mechanisms fully. Plant colonization rates of AMF and DSEs vary substantially, primarily influenced by growth age and environment [34, 35, 36, 37]. For the triennial samples, AMF colonization rates ranged from 13.03 to 39.48% with a mean of 27.02%, similar to the rate of 27.54% reported in *Longjing* tea root [38]. Jin et al. [39] reported a significant 70% increase in the AMF colonization rate of *Solidago canadensis* in dry habitats over time, consistent with our observations of increased AMF colonization in BNP, WSP and YCP samples. However, AMF colonization rates declined with the plant age in HHP samples, potentially due to competition fro*m* other microorganisms or excessive application of fertilizers and pesticides.

Relative to the DSE colonization rate of 21.25% in *Artemisia deversa* [40], annual samples of *A. villosum* from five sampling plots had lower DSE colonization rates, ranging from 0 to 9.38% with a mean value of 3.11%. This difference may be attributable to nutrition patterns and growth environment [32, 38]. Nevertheless, the mean DSE colonization rate increased significantly with plant age, reaching 6.15%. Moreover, AMF and DSEs colonization rates in the PEP-1 sample were higher than in other samples, consistent with the hypothesis of synergistic interaction between AMF and DSEs [33]. This finding aligns with studies by Della Mónica et al. [33] and Ranelli et al. [41], which reported positive correlations between AMF and DSEs colonization in *Trifolium repens* and *Achnatherum lettermanii*, linking both fungi to phosphorus utilization and uptake.

Fungal taxa showed no significant differences among *A. villosum* samples from the five plots. The dominant phyla were Ascomycota, Basidiomycota, and Glomeromycota, mirroring the root fungi composition of *Casuarina equisetifolia* [36]. Fungi from three phyla represent the most abundant groups colonizing plant roots [42]. At the order level, Hypocreales, Chaetothyriales, and Helotiales occurred in all samples, along with the families Cordycipitaceae and Cladosporiaceae, previously identified in plants like *Fragaria × ananassa* [43] and *Glycine max* [44] for their biocontrol potential. Hypocreales are widely commercialized as biofungicides, and Cladosporiaceae species exhibitpotential for white rust control [45, 46]. The antimicrobial genus *Phoma*, saprophytic genera *Acremonium* and *Myrothecium* and the biocontrol genus *Trichoderma* were detected as core genera herein, contributing to rhizosphere microstructure improvement [47, 48]. *Beauveria* emerged as a dominant genus in *A. villosum* roots, which not only induces no plant diseases but also confers insect pest resistance and facilitates exchange nitrogen in the host [49]. Therefore, the functions of *Beauveria* require dialectical consideration. Importantly, AMF and DSEs constituted the core components within the *A. villosum* root system, reflected in the colonization rates. *Glomus* (AMF) and *Exophiala*, *Cladosporium*, and *Cladophialophora* of DSEs were identified as core genera. According to previous studies, AMF and DSEs were proven to be the dominant fungal communities in *Glycyrrhiza uralensis*, *Ferula sinkiangensis* and *Tinospora cordifolia*, e.g., *Glomus*, *Diversispora* and *Cladosporium* [50, 51, 52]. The colonization by these diverse mycorrhizal fungi may establish a complex network within the root system, crucial for plants growth maintenance and disease resistance.

The alpha diversity results showed that neither species richness nor diversity indices differed significantly with plant age; rather, observed variations were primarily attributable to spatial heterogeneity. Similar to *C. equisetifolia* endophytic fungal communities reported by Huang et al. [36], diversity indices showed no significant variation across forest ages. This phenomenon may be attributed to the fact that plant root endophytic fungal diversity is largely influenced by local biotic and abiotic influences [53], e.g., soil properties as well as human disturbances both cause changes in root microbial diversity [54, 55]. Notably, fungal diversity was consistently highest in the HHP plot among the five, which may be the result of multiple factors. However, the abundance of AMF decreased in triennial samples of HHP, suggesting that it may be related to the change in soil nutrient composition over time and may require the involvement of other microorganisms for adjustment.

Community composition analysis indicated minimal variation across plant ages, identifying geographic location as the primary driver of community divergence. Random forest analysis identified specific fungal taxa partially responsible for compositional differences. Consistent with Zhong et al. [56], *Nigrospora* served as a biomarker or key taxon herein. It was found that the *Diversispora* (AMF) was involved in the growth promotion of *Chrysanthemum morifolium* under salt stress [57], and significantly enriched in annual samples, implying a pivotal role during this stage. In contrast, *Aspergillus* and *Cladosporium* (DSEs) were enriched in triennial samples and exhibited significantly from annual *A. villosum* samples. These genera have been reported to confer heavy metal resistance and may contribute to maintaining and promoting plant growth [58, 59]. Colonization surveys and molecular diversity analyses collectively underscore that AMF and DSEs influence the evolution of plant rhizosphere endophytic fungal communities and undergo distinct structural changes driven by spatial and temporal factors.

While bacteria and non-endophytic fungi critically influence rhizosphere ecology and plant growth through microbe-microbe interactions [60, 61], this study focused exclusively on endophytic fungal colonization/diversity in *A. villosum*. We acknowledge this limitation in assessing the full plant-microbe symbiont system. Future work will prioritize investigating: (1) endophyte-mediated bacterial recruitment, and (2) synergistic mechanisms of microbial consortia in enhancing medicinal plant growth, stress resilience, and bioactive compound accumulation.

## Conclusions

Through fungal colonization rate surveys and high-throughput sequencing, we revealed differences in both colonization rates and community structures of EF (mainly AMF and DSEs) in *A. villosum* roots across varying locations and growth ages. Dominant taxa included, *Glomus* (AMF) and the DSE genera *Exophiala*, *Cladosporium*, and *Cladophialophora*, all exhibiting increased abundance with host maturation. Random forest analysis identified *Nigrospora* and *Cladosporium* as EF biomarkers for annual and triennial samples, respectively, suggesting age-specific functional roles in growth dynamics. Taken together, our study reveals that spatial heterogeneity drives divergent root fungal communities in *A. villosum*, while plant age selectively enriches key beneficial EF. Given the significant economic value of *A. villosum* as a key traditional Chinese medicinal plant, our findings provide an important foundation for the development of specialized fungal fertilizers and their application in its ecological cultivation.

## Supplementary Information

Below is the link to the electronic supplementary material.

Supplementary Material 1

## Data Availability

No datasets were generated or analysed during the current study.
