# Supplementary material for: Diversity and dynamics of fungal endophytes in the roots of Amomum villosum lour. Under different areas and growth ages
Source: BMC Microbiol. 2025 Aug 26;25:550. doi: 10.1186/s12866-025-04332-6 (PMC12379508; doi:10.1186/s12866-025-04332-6)
Supplement: Supplementary file 1 — Supplementary Material 1 [file 12866_2025_4332_MOESM1_ESM.zip › Revised Supplementary_Material 2024 11/Supplementary Tables .docx]

**Table S1.** The geographic coordinates of sampling points and agro-meteorological data

| **Sample plots** | **Longitude (N)** | **Latitude (E)** | **Annual mean temperature (℃)** | **Annual rainfall (mm)** |
| --- | --- | --- | --- | --- |
| Honghe (HHP) | 103.289 | 22.988 | 22 | 828.7 |
| Wenshan (WSP) | 104.897,942 | 23.31,727 | 21 | 1,271.1 |
| Puer (PEP) | 102.194,939 | 22.491,903 | 24 | 867.1 |
| Xishuangbanna (BNP) | 101.005,638 | 22.093,481 | 29 | 587.8 |
| Yangchun (YCP) | 111.493,317 | 22.31,954 | 28 | 1,456.5 |

**Table S2**. Sequencing data quality assessment

| **Sample ID** | **Raw PE** | **Raw tags** | **Clean tags** | **Effective tags** | **Effective rates (%)** | **Average length (nt)** | **Q30 (%)** |
| --- | --- | --- | --- | --- | --- | --- | --- |
| BNP1_1 | 36,750 | 33,605 | 33,147 | 32,020 | 87.13% | 340 | 94.74% |
| BNP1_2 | 34,442 | 33,029 | 32,668 | 31,802 | 92.33% | 339 | 94.12% |
| BNP1_3 | 32,571 | 31,127 | 30,684 | 29,818 | 91.55% | 339 | 93.08% |
| HHP1_1 | 32,773 | 31,859 | 31,307 | 30,082 | 91.79% | 343 | 93.83% |
| HHP1_2 | 33,867 | 31,353 | 30,910 | 29,695 | 87.68% | 343 | 94.04% |
| HHP1_3 | 33,580 | 32,726 | 32,254 | 31,225 | 92.99% | 343 | 94.74% |
| PEP1_1 | 34,564 | 31,658 | 31,293 | 30,482 | 88.19% | 343 | 94.46% |
| PEP1_2 | 36,239 | 34,639 | 33,984 | 32,940 | 90.90% | 347 | 94.24% |
| PEP1_3 | 32,595 | 31,825 | 31,459 | 30,254 | 92.82% | 348 | 94.53% |
| WSP1_1 | 37,103 | 34,908 | 34,241 | 33,032 | 89.03% | 341 | 94.17% |
| WSP1_2 | 35,958 | 34,935 | 34,508 | 33,603 | 93.45% | 342 | 93.13% |
| WSP1_3 | 40,742 | 39,452 | 38,943 | 37,552 | 92.17% | 342 | 94.23% |
| YCP1_1 | 39,216 | 35,880 | 35,313 | 34,299 | 87.46% | 341 | 93.15% |
| YCP1_2 | 32,569 | 31,459 | 30,858 | 29,768 | 91.40% | 341 | 94.10% |
| YCP1_3 | 33,287 | 32,593 | 31,980 | 30,825 | 92.60% | 341 | 94.11% |
| BNP3_1 | 32,759 | 31,352 | 30,941 | 29,929 | 91.36% | 339 | 94.88% |
| BNP3_2 | 35,050 | 32,718 | 32,309 | 31,433 | 89.68% | 352 | 94.69% |
| BNP3_3 | 34,487 | 31,398 | 31,036 | 29,952 | 86.85% | 338 | 94.81% |
| HHP3_1 | 33,220 | 32,436 | 31,842 | 30,590 | 92.08% | 344 | 94.17% |
| HHP3_2 | 37,866 | 34,584 | 34,079 | 32,910 | 86.91% | 343 | 94.04% |
| HHP3_3 | 33,084 | 30,831 | 30,522 | 29,447 | 89.01% | 340 | 94.63% |
| PEP3_1 | 32,843 | 31,810 | 31,383 | 30,275 | 92.18% | 339 | 93.79% |
| PEP3_2 | 32,724 | 31,107 | 30,789 | 29,582 | 90.40% | 339 | 93.10% |
| PEP3_3 | 33,387 | 32,116 | 31,525 | 30,292 | 90.73% | 338 | 94.84% |
| WSP3_1 | 42,430 | 38,735 | 38,165 | 37,207 | 87.69% | 340 | 93.57% |
| WSP3_2 | 37,383 | 34,941 | 34,441 | 33,400 | 89.35% | 341 | 94.48% |
| WSP3_3 | 35,474 | 32,738 | 32,151 | 31,051 | 87.53% | 342 | 94.99% |
| YCP3_1 | 33,409 | 32,544 | 32,029 | 31,135 | 93.19% | 337 | 93.52% |
| YCP3_2 | 36,726 | 33,806 | 33,440 | 32,299 | 87.95% | 336 | 93.72% |
| YCP3_3 | 31,592 | 30,518 | 29,990 | 28,853 | 91.33% | 335 | 93.91% |

**Table S3**. Richness and diversity of endophytic fungi in different samples

| Sample name | Observed | Chao1 | ACE | Shannon | Simpson | Coverage | PD |
| --- | --- | --- | --- | --- | --- | --- | --- |
| PEP1_1 | 141.000 | 158.103 | 166.047 | 1.071 | 0.340 | 0.999 | 24.707 |
| PEP1_2 | 179.000 | 207.700 | 211.153 | 1.939 | 0.745 | 0.999 | 28.182 |
| PEP1_3 | 188.000 | 205.000 | 211.584 | 2.024 | 0.754 | 0.999 | 29.797 |
| HHP1_1 | 279.000 | 295.500 | 299.341 | 3.477 | 0.926 | 0.999 | 43.259 |
| HHP1_2 | 288.000 | 310.658 | 314.120 | 3.295 | 0.904 | 0.999 | 42.433 |
| HHP1_3 | 284.000 | 306.677 | 308.251 | 3.483 | 0.929 | 0.999 | 43.883 |
| YCP1_1 | 141.000 | 171.565 | 171.764 | 1.110 | 0.381 | 0.999 | 27.522 |
| YCP1_2 | 187.000 | 190.889 | 193.924 | 2.863 | 0.855 | 1.000 | 33.493 |
| YCP1_3 | 180.000 | 206.464 | 212.075 | 1.700 | 0.601 | 0.999 | 36.351 |
| BNP1_1 | 118.000 | 136.125 | 137.925 | 0.774 | 0.260 | 0.999 | 22.744 |
| BNP1_2 | 116.000 | 124.053 | 126.034 | 1.221 | 0.587 | 0.999 | 23.391 |
| BNP1_3 | 146.000 | 165.333 | 165.860 | 1.376 | 0.438 | 0.999 | 25.395 |
| WSP1_1 | 208.000 | 226.529 | 236.363 | 2.408 | 0.721 | 0.999 | 33.262 |
| WSP1_2 | 175.000 | 208.158 | 197.690 | 1.839 | 0.565 | 0.999 | 30.263 |
| WSP1_3 | 177.000 | 197.583 | 211.182 | 2.212 | 0.705 | 0.999 | 27.798 |
| PEP3_1 | 151.000 | 178.556 | 175.896 | 2.385 | 0.804 | 0.999 | 31.885 |
| PEP3_2 | 148.000 | 185.435 | 188.686 | 2.191 | 0.763 | 0.999 | 31.898 |
| PEP3_3 | 137.000 | 155.125 | 160.091 | 2.380 | 0.848 | 0.999 | 30.356 |
| HHP3_1 | 183.000 | 214.714 | 219.473 | 3.095 | 0.865 | 0.999 | 32.562 |
| HHP3_2 | 188.000 | 231.158 | 217.887 | 2.660 | 0.823 | 0.999 | 32.598 |
| HHP3_3 | 132.000 | 159.000 | 153.321 | 2.512 | 0.848 | 0.999 | 25.111 |
| YCP3_1 | 149.000 | 157.143 | 158.909 | 1.698 | 0.650 | 0.999 | 24.280 |
| YCP3_2 | 183.000 | 218.150 | 215.673 | 1.853 | 0.650 | 0.999 | 31.969 |
| YCP3_3 | 211.000 | 249.607 | 255.487 | 1.766 | 0.645 | 0.998 | 38.173 |
| BNP3_1 | 154.000 | 187.682 | 190.458 | 2.397 | 0.772 | 0.999 | 36.067 |
| BNP3_2 | 188.000 | 201.316 | 199.580 | 2.578 | 0.861 | 0.999 | 32.763 |
| BNP3_3 | 179.000 | 186.028 | 194.448 | 2.419 | 0.783 | 0.999 | 34.569 |
| WSP3_1 | 161.000 | 198.840 | 205.069 | 2.329 | 0.777 | 0.998 | 29.713 |
| WSP3_2 | 225.000 | 244.524 | 256.759 | 2.797 | 0.809 | 0.999 | 34.331 |
| WSP3_3 | 227.000 | 263.029 | 273.914 | 2.767 | 0.802 | 0.998 | 34.351 |
