# Supplementary material for: Diversity and dynamics of fungal endophytes in the roots of Amomum villosum lour. Under different areas and growth ages
Source: BMC Microbiol. 2025 Aug 26;25:550. doi: 10.1186/s12866-025-04332-6 (PMC12379508; doi:10.1186/s12866-025-04332-6)
Supplement: Supplementary file 1 — Supplementary Material 1 [file 12866_2025_4332_MOESM1_ESM.zip › Revised Supplementary_Material 2024 11/Supplementary figure legend.docx]

**Figure S1**. Relative abundance of dominant (>0.1%) fungal taxa (phyla, orders, families and genera) in *A. vilosum* rhizosphere of different ages and sample plots. The common dominant class was Sordariomycetes (81.88 and 71.77%%, one-year/three-year), in which Dothideomycetes accounted for 7.61% more in the triennial samples than in the annual samples. Pleosporales (13.00 and 19.53%, one-year/three-year) and ectomycorrhizal fungi (EMF) Hypocreales (66.63 and 50.53%, one-year/three-year) were the common dominant orders in annual and triennial samples, respectively. Cordycipitaceae (51.66 and 15.92%, 1-year/3-year) and Nectriaceae (8.28 and 7.15%, one-year/three-year) were the codominant families.
