# Supplementary figures and images for: Diversity and dynamics of fungal endophytes in the roots of Amomum villosum lour. Under different areas and growth ages
Source: BMC Microbiol. 2025 Aug 26;25:550. doi: 10.1186/s12866-025-04332-6 (PMC12379508; doi:10.1186/s12866-025-04332-6)

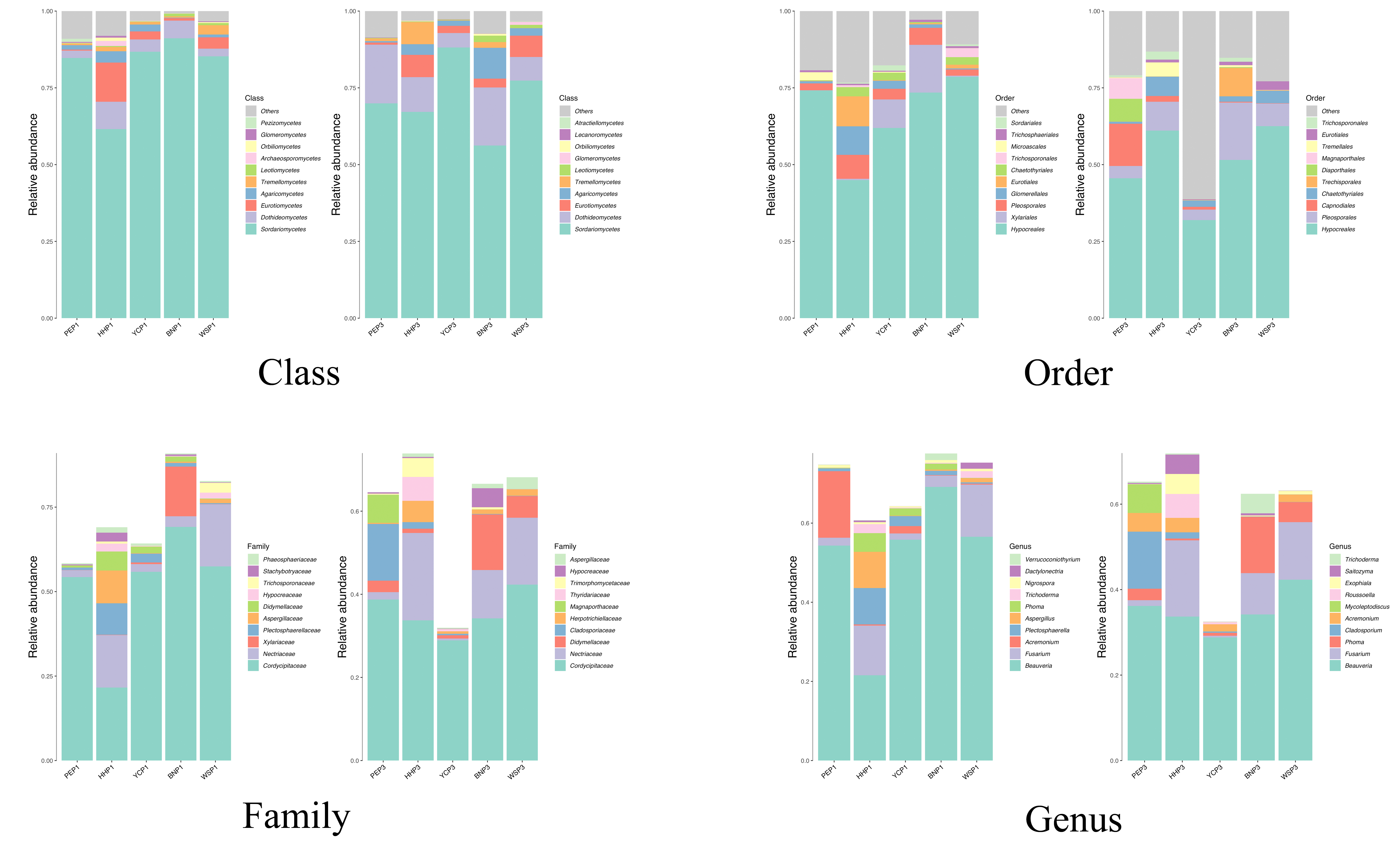

Supplement: Supplementary file 1 — Supplementary Material 1 [file 12866_2025_4332_MOESM1_ESM.zip › Revised Supplementary_Material 2024 11/Fig. S1.tif]
